# Eye-Catching Odors: Olfaction Elicits Sustained Gazing to Faces and Eyes in 4-Month-Old Infants

**DOI:** 10.1371/journal.pone.0070677

**Published:** 2013-08-28

**Authors:** Karine Durand, Jean-Yves Baudouin, David J. Lewkowicz, Nathalie Goubet, Benoist Schaal

**Affiliations:** 1 Developmental Ethology and Cognitive Psychology Group, Center for Smell, Taste and Food Science, Dijon, France; 2 Department of Psychology, Florida Atlantic University, Boca Raton, Florida, United States of America; 3 Department of Psychology, Gettysburg College, Gettysburg, Pennsylvania, United States of America; 4 Institut Universitaire de France, Paris, France; Lancaster University, United Kingdom

## Abstract

This study investigated whether an odor can affect infants' attention to visually presented objects and whether it can selectively direct visual gaze at visual targets as a function of their meaning. Four-month-old infants (n = 48) were exposed to their mother's body odors while their visual exploration was recorded with an eye-movement tracking system. Two groups of infants, who were assigned to either an odor condition or a control condition, looked at a scene composed of still pictures of faces and cars. As expected, infants looked longer at the faces than at the cars but this spontaneous preference for faces was significantly enhanced in presence of the odor. As expected also, when looking at the face, the infants looked longer at the eyes than at any other facial regions, but, again, they looked at the eyes significantly longer in the presence of the odor. Thus, 4-month-old infants are sensitive to the contextual effects of odors while looking at faces. This suggests that early social attention to faces is mediated by visual as well as non-visual cues.

## Introduction

Human infants have keen noses. This is evident from findings indicating that arbitrary odorants can elicit autonomic reactions or motor responses in infants ([1]–[2]; reviewed in [3]–[4]). It is also evident from studies assessing the role of natural odorants or of extraneous odorants familiarized during nurturing interactions. The latter studies have found that olfaction promotes a range of early regulatory functions. Specifically, odors can modulate infants' arousal states [5]–[6], delay, reduce, or terminate distress and attenuate its physiological correlates [7]–[8], elicit directional head or body movements [5], [9]–[11], stimulate breathing and oral-lingual actions [12]–[14], control ingestive behavior [15]–[16], and initiate the affective tagging of objects/contexts to be approached, avoided, or ignored [17]. In addition, when newborns are exposed to their mother's breast odor, they display longer episodes of eye opening as compared to the same situation with no odor [8].

Despite this large body of evidence on the effects of odors on infant responsiveness, little is known about the possibility that olfaction may interact with other sensory modalities and about the behavioral consequences of such interactions [18]. It is likely that such intermodal effects occur because the odors experienced in the context of infant-mother relations continue to modulate infants' exploratory or hedonic responses for weeks to months after learning [19]–[21]. In addition, it has been found that 3-month-olds can learn to control the movements of a mobile by kicking and that they better remember doing this the next day when the same odor is present than when an unfamiliar odor is delivered [22]. Thus, infants pick up contextual odor information and rely on it in the control of their actions. Infants can also associate an arbitrary odor/flavor with an arbitrary object and can later remember the odor as one of that object's properties [23]–[24]. In sum, despite the common belief that infants are overwhelmed by inputs in other modalities as well as inputs from their own motor activity (*e.g.*, [25]), findings to date suggest that olfaction plays an important role by itself and in interaction with other sensory modalities during early development.

Vision is a modality that is most likely to interact with olfaction. From birth, infants orient preferentially to face-like patterns as compared to arbitrary visual stimuli of equivalent complexity [26]–[28] and newborns can discriminate different face-like configurations and different pictures of unfamiliar real faces [29]. Furthermore, newborns are able to learn the individual properties of their mother's face and display a preference for her over an unfamiliar female's face [30]–[33]. Such early face recognition abilities are robust enough that they are observed even under conditions of partial occlusion [34], rotation of viewpoint [35], or masking of inner/outer facial features [36]. Despite the early abilities to learn, discriminate and remember faces, these abilities improve as infants grow and gain perceptual experience. Moreover, their specific preferences change as they gain in perceptual sophistication. For example, the preference for mother's over an unfamiliar female's face before two months of age reverses to a preference for the unfamiliar female's face at five months of age [37].

When young infants look at adult faces, they look mostly at the eyes. Even though during the first weeks of life, infants mainly concentrate on external features of faces (chin and hairstyle) and when they scan the internal features of a face they look longer at the eye region than at the mouth [36]–[40]. Infants also look longer at faces which eyes are directed straight ahead than at faces with eyes averted [41]. This eye-contact effect constitutes a basic interactive element in human visual exchanges throughout the life-span [42], and already during the first months of life, the perception of direct gaze modulates cortical activation [41] and facilitates the learning of faces [43]. Thus, early eye-to-eye contact may promote visual attention and the learning of facial features of individuality and emotional states.

With a few notable exceptions [23]–[24], the interaction between olfaction and vision has not been investigated in early development. Despite this, an increasing number of studies has shown that smell can have a strong effect on visual perception, especially when it comes to humans' perceptual appraisal of the environment. Specifically, in adults, olfaction has been found to influence higher cognitive functions [44]–[46], such as memory formation [47], semantic priming [48], and the unconscious driving of affects and actions, either by direct, immediate influence on behavior or by the mediation of underlying mood and attitude changes [45], [49]–[50]. With specific regard to faces, adults' response has been shown to be modulated by olfaction. For example, the affective valence carried by different odorants influences likeability judgments of concurrently presented faces [51]. It is noteworthy that this effect is best exerted when the odor is not accessible to conscious awareness [52]. Furthermore, a study on the co-processing of odors and faces has indicated that subconscious odor inputs improve the encoding of faces and the formation of related memory [53].

The types of odor-vision interactions that have been studied in adults have not been investigated in early human development. Nonetheless, infant perception of faces is known to be influenced by co-occurring non-visual stimuli (*e.g.*, voice: [40], [55]–[57]; touch: [58]; kinesthesis: [59]). Therefore, it is likely that olfaction and vision interact in early development as well [18]. There are a number of *a priori* reasons to posit this possibility. From birth onwards, infants have considerable exposure to their mother's multiple and concurrent sensory attributes, which are experienced repeatedly in the highly arousing and rewarding conditions created by nursing, swaddling, and affective touch. The result of such exposures is that infants experience temporally and spatially distributed touches, smells, tastes, faces, and voices that give rise to representations of specific people. For example, the nursing-related eye-opening behavior noted in newborns [8] increases over the first weeks [60], so that nursing creates privileged periods during which infants concomitantly perceive, among other stimuli, their mothers' odor and face. Given that odors can rapidly gain affective meaning by association with nurturance [11], [20], specific olfactory experience can be associated with specific visual experience. Conversely, being exposed to an eye-to-eye gazing face is highly rewarding from the neonatal period onwards [42], and may alter the hedonic value of any co-occurring odor.

Regardless of the direction of influence, at this point the most interesting question is whether olfaction and vision interact and, specifically, whether specific odors can affect infants' visual behaviors. As a result, the present study investigated whether and how the duration and pattern of looking at a visual scene is influenced by a co-occurring odor in 4 month-old infants. Infants were exposed to paired visual stimuli, consisting of a social object (the static face of an unfamiliar female) and a non-social object (a picture of a car), and a social odor (the body odor of their mother). Several outcomes were hypothesized. First, regardless of discriminative responses to the social vs. nonsocial meaning of the pictures, and based on findings showing that the mother's odor influences the newborn's overt and covert arousal (oral activity, respiratory rate) and visual behavior (eye opening) [8], [13], we expected that the odor may affect the infants' arousal and visual behavior in two complementary ways. Either, the mother's body odor may produce general arousal, leading infants to indiscriminately increase their attention to, and exploration of, both objects in the visual scene, or the combination of olfactory and visual stimulation may induce higher arousal which would also lead to greater attention to both visual objects relative to the attention they might exhibit to the visual stimuli in the absence of the odor. Second, 4-month-olds may look more at pictures of conspecifics than at pictures of arbitrary objects [61]. Therefore, infants may look longer at the social picture in the presence of the social odor, either because they perceive more congruence between a female face and a female odor or because they detect incongruence between the familiar mother's body odor and an unfamiliar female face. Otherwise, a social odor may induce more general social expectations or the desire to engage in social interaction. Accordingly, if social odors prompt a desire for social interaction and communication, the infants' pattern of looking at the face should differ in the “odor present” and the “odor absent” conditions. If the mother's body odor was previously learned as contingent with proximal interactions, the mere fact of diffusing it should intensify the infant's search for the visual stimulus that is normally associated with interaction. Thus, we predict that infants should look longer at the social stimulus (the face) in the social odor condition than in the control odor condition.

## Methods

### Participants

This study was approved by the CERES (Conseil Ethique pour les Recherches en Santé) of Paris-Descartes University and conducted according to the principles stated in the Declaration of Helsinki. Four month-old infants were recruited through the local birth registry. Parents were contacted by phone by an assistant who explained the aims and methods of the study. If they agreed to let their infant participate, they were sent an informed consent sheet, as well as the material for sampling their body odor (see below). The experiment was explained in fuller detail when the parents then came to the baby laboratory. Ultimately, all parents gave written consent to let their infant take part in the study and were physically present during the experimental session.

Seventy-nine Caucasian infants were tested. Due to non-compliance (agitation, discomfort), technical complications (inadequate eye capture or calibration of eye gaze by the eye tracking system), or the failure to satisfy the inclusion criteria for appropriate visual behavior (see below), 31 infants were excluded from further analyses (17 and 14 in the odor and control groups, respectively). The final sample consisted of 48 infants (26 females; mean age ± SD: 123±2.6 days; age range: 117–128 days). At the time of testing, 19 infants were exclusively breast-fed (8 and 11 in the odor and control groups, respectively). All infants and their mothers were in excellent health conditions on the day of testing and the mothers were in good health during the several days before (during maternal body odor sampling). The group of infants exposed to the worn T-shirt and the group of infants exposed to the unworn control T-shirt were comparable in terms of age (mean: 123 *vs.* 123 days; range: 117–128 *vs.* 118–128 days, respectively) and sex-ratio (males/females: 12/12 *vs.* 10/14, respectively).

### Stimuli

#### Visual Stimuli

The visual stimuli consisted of social stimuli (feminine faces) and non-social stimuli (cars). Twelve color photographs of Caucasian women's faces and of cars were selected from the authors' personal image database or from the Internet. Each female face was photographed in a frontal pose, with an emotionally neutral facial expression and with gaze directed at the perceiver. The faces differed in hair color and style. Female faces were chosen because they seem to be more attractive for young infants than male faces [62]. The pictures of the faces were paired with pictures of cars, often considered as having face-like properties [63]. Each car differed in shape and color (*e.g.*, white Peugeot, red Renault) and was presented in frontal or 3/4 pose. The dimensions of the photographs were 500×500 pixels, corresponding on the screen to a face of 17 cm in height and 10 cm in width and, for the car, to an object of 11 cm in height and 18 cm in width. Each photograph subtended a visual angle of 19 degrees.

**Figure 1:**
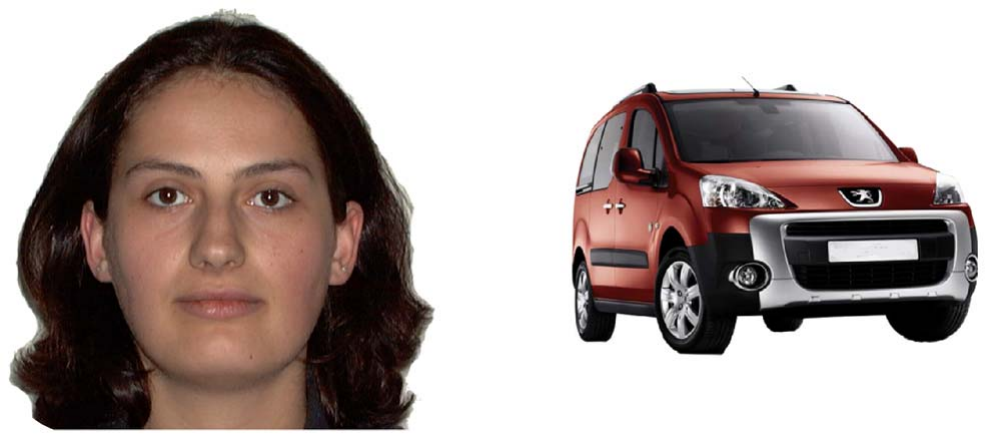
The face and car stimuli used in the visual exploration task (different faces and cars were presented within and between subjects). The subject on the photograph has given written informed consent, as outlined in the PLOS consent form, to publish her picture.

From these photographs, 12 pairs of visual stimuli were created, each composed of a female face presented side-by-side against a car (Figure 1) on a white-background screen. Each face was paired with a different car. The cars were selected to be variable in color and shape with the intention of maintaining infants' attention in the car during the four trials of the test while, at the same time, avoiding the possibility of ceiling effects for face-directed attention. The visual stimuli were displayed by the Clearview software (Tobii's Clearview AVI presentation software, Stockholm, Sweden) designed for the eye movement-tracking system (Tobii 2150, Tobii Technology, Stockholm, Sweden). The different pairs of pictures were counterbalanced between infants and between olfactory conditions, so that each pair appeared as frequently during the different trials in each odor condition

#### Odor Stimuli

Subjects participated in one of two conditions, one involving a familiar odor and one involving a control odor. The familiar odor was the mother's body odor collected on a t-shirt. A t-shirt (100% cotton) was sent (enclosed in a paper bag itself enclosed in a zip-locked hermetic plastic bag) to the mother in the week preceding the testing of her infant. The t-shirt was worn by the mothers for the 3 consecutive nights preceding their visit to the laboratory. Over this period, they were asked to wear the t-shirt on their skin without using perfume and in refraining as much as possible to shower with odorous soap. During the days, they were instructed to put the t-shirt into the paper bag itself put into the hermetically locked plastic bag that could be left at ambient temperature but far from any heating device. The control stimulus consisted of an unworn t-shirt that was conserved in both bags in the same conditions. Before the experiment began, all t-shirts were laundered (using Paillettes soap, Le Chat, Marseille, France) and dried in the same standard conditions in the laboratory.

After test completion, the t-shirts were stored to be later submitted to descriptive odor assessment. The storage of the t-shirts was made in a devoted congelator for maximum 4 weeks in conditions (−20°C) known to satisfy conservation of their odor properties [86]. Nine non-smoking adult judges (mean age: 32.7 years, range: 25–40 years; 5 females) assessed 23 worn and 9 unworn control t-shirts along several dimensions. The worn t-shirts belonged to the mothers of only the infants who contributed usable data. They were required to smell one by one the t-shirts (introduced into plastic bags) and to rate on 7-point Likert scales how *intense* their odor was [from 1 (unnoticeable) to 7 (extremely intense)], whether it carried a note of *perfume* [from 1 (unnoticeable) to 7 (extremely perfumed)] and/or of *sweat* [from 1 (unnoticeable) to 7 (extremely sweaty)], how much they found the odor *arousing/stimulating* [from 1 (arousing/stimulating) to 7 (calming/appeasing)], and how *pleasant* the odor was [from 1 (extremely pleasant) to 7 (extremely unpleasant]. The mean scores obtained for the worn t-shirts and the unworn t-shirts were respectively 5.11±0.93 *vs.* 4.26±0.96 for the rating of intensity, 3.72±1.04 *vs.* 3.12±0.72 for the rating of perfumed note, 3.69±1.46 *vs.* 2.87±0.69 for the sweat note rating, 4.74±0.55 *vs.* 5.06±0.48 for the rating of arousing effect, and 4.99±1.31 *vs.* 5.48±0.95 for the rating of pleasantness. Although it might be surprising that unworn control t-shirts were given relatively high ratings, it must be noted that all t-shirts, even new or freshly laundered ones, have an inherent and unavoidable smell that is due to the fabric and the way it is treated. However, three nights of wearing the t-shirts appeared sufficient to render them significantly differentiable from unworn control t-shirts in terms of sweaty odor and subjective intensity ratings [t(30) = 2.15 and 2.12, respectively, p<0.05 in both cases], but not in terms of perfume odor, arousing effect and pleasantness ratings.

### Experimental setting and procedure

The infants were brought into the Baby Lab of the Centre for Smell, Taste and Food Science. The testing took place in an area enclosed by partitions. All windows were occluded and the lights were switched off during the experiment to limit visual distraction. The room was well aired between testing sessions and experimenters were instructed to refrain from using perfume or drinking coffee before the experiments. The infants were securely and comfortably seated in a baby-seat in semi-reclining position. Their face was positioned facing the screen of the eye-tracking system at a distance of about 60 cm and a video camera at a distance of about 100 cm. The parents sat behind and far enough (>2.5 m) from their infants to avoid perceiving personal odor (the parents were asked not to use perfume on the day of the experiment). During the experiment, the parents were instructed not to intervene (in speaking or coming near to the infant).

The infants' eye movements were followed for each eye using the eye movement-tracking system. The experiment began when the infant was placed in the seat. Each infant was randomly assigned either to the worn or to the control unworn t-shirt. Depending on group assignment, the worn or the control t-shirt was affixed on the infants' upper chest, right under the chin, so that they could inhale its effluvium. The t-shirts were folded so that infants would be exposed to the axillary, breast, and neck regions. All of these body locations have been shown to produce odorous substrates to which infants are reactive [64].

Then, each infant participated in two experimental phases: the calibration of the eye-tracker and the experiment itself. The standardized calibration of the eye-tracker consisted of presenting on a white screen a cartoon figure that moved while it emitted a rattle sound. When the infant looked at it for at least 1 s, the figure moved to another position on the screen and remained in that position until fixated again for at least 1 s. This was repeated for up to 9 positions covering the whole surface of the screen (center, 4 corners, and 4 intermediate positions close to the screen borders). If the eye-tracker did not find the eyes for one or more of these positions, the calibration procedure was run again for the screen positions in which the eyes were not captured. In case the system did not find the eyes for some positions after 3 such calibration trials, the test procedure was run anyway. The infants were retained for further analyses only if their gaze was detected for at least 5 calibration positions. All 48 infants passed the calibration phase successfully.

After calibration, the experimental phase began. The two groups of infants were shown the paired visual stimuli while their looking behavior was recorded. The test consisted of 4 trials during which each pair of visual stimuli was presented twice consecutively, before another pair of stimuli appeared twice consecutively. The lateral position of the two pictures was counterbalanced across infants and trials. Further, the different pairs of pictures were counterbalanced between infants and between olfactory conditions, so that each pair appeared as frequently during the different trials in each odor condition. Each trial lasted 30 s with an inter-trial period of 1 s during which a blue screen appeared.

### Dependent variables

The eye-tracker computed the amount of looking at the face and the car for each infant and, based on these measures, the total duration of looking was calculated across the four trials in both groups. Further variables involved which target was fixated first, latencies to reach any visual target, and amount of switching between targets. To determine how much time infants spent gazing at the eyes, nose, mouth and other face regions, we delimited four areas of interest (AOI) on the face, one around both eyes, one around the nose, one around the mouth, and one for the other regions of the face (as shown in the insert on figure in Results). The spatial coordinates of infants' point-of-gaze (POG) were monitored with the eye-tracker and whenever a POG fell into an AOI, it was considered a look in that particular AOI. The total duration of looking in each AOI was timed by the eye-tracker. Thus, the time of visual fixation over a given AOI includes both fixations and saccades that infants have directed within this AOI. When not stated otherwise, all data are presented with standard deviations (SD).

## Results

Preliminary analyses of variance (ANOVA) indicated that there were no significant effects of infants' gender and feeding status (breast- vs. bottle) on looking times. Accordingly, these factors were not taken into account in the following analyses.

### Target-related visual behavior

We assessed whether the mother's body odor affected infants' visual exploration of the pictures depicting an unfamiliar female face and a car. A 2 (odor group) by 2 (visual stimuli) by 2 (pairs) by 2 (trials) mixed ANOVA on looking time, with odor as a between-subjects variable, and visual stimuli, pairs, and trial as within-subjects variables, yielded a significant main effect of visual stimulus [F(1,46) = 41.49; *p*<0.0001]. Thus, regardless of odor condition, infants looked longer at the picture of the female face than at the car picture (mean ± SD: 11.8±5.6 *vs.* 6.3±4 s).

**Figure 2:**
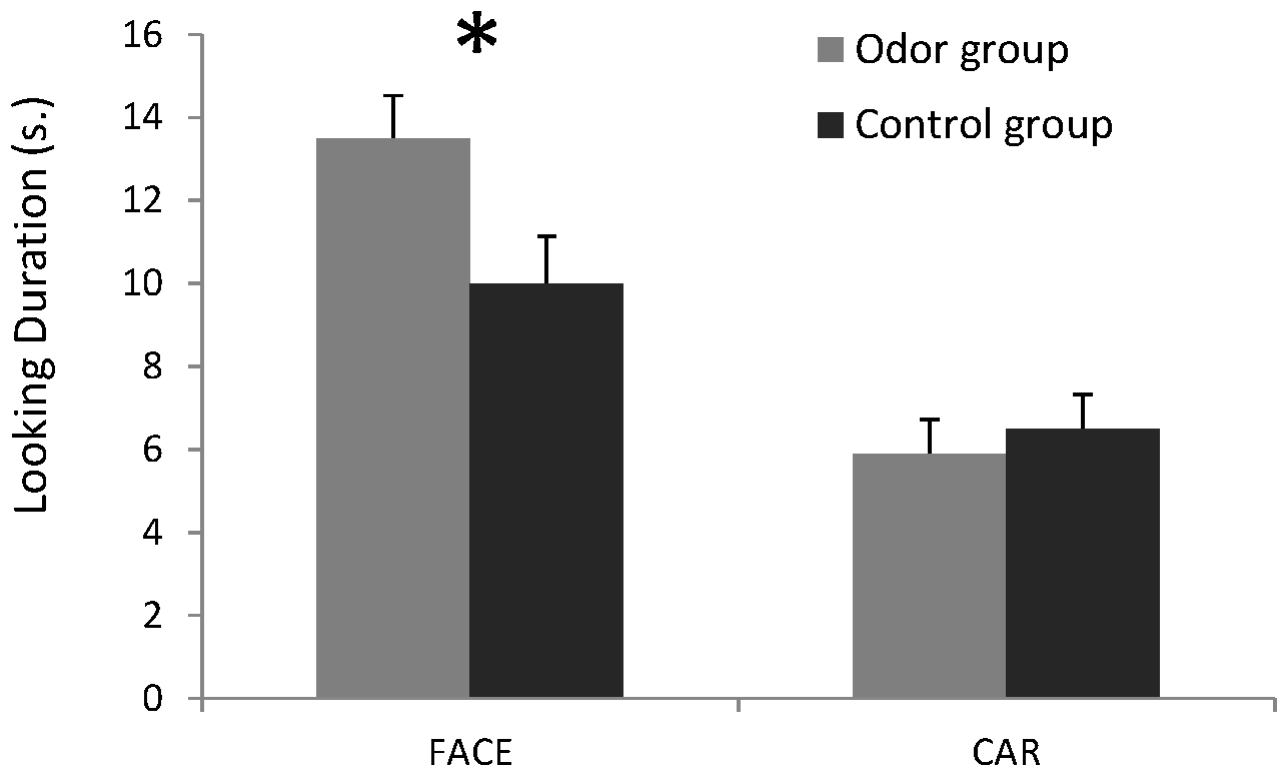
Mean looking duration (in seconds) to the face and at the car in a group of infants concurrently exposed to a t-shirt carrying their mother's odor (odor group) and in a group of infants exposed to an unworn control stimulus (control group). Error bars represent the standard errors of the mean (SEM). _*_: p<0.05.

In addition to the main effect of visual stimulus, we found that the odor condition interacted significantly with the type of visual stimulus presented [F(1,46) = 5.60; *p*<0.025]. That is, even though infants exhibited an overall preference for the female face, the group of infants exposed to the worn t-shirt looked longer at the female face than the group of infants exposed to the control t-shirt: the odor group looked nearly twice as long at the female face than at the car picture (mean looking time per trial: at Face = 13.5±5 *vs.* Car = 6±4 s) as compared to the control group (Face: 10±5.6 *vs.* Car = 6.5±4 s) (Figure 2). Planned comparisons indicated that, while exposed to their mother's body odor, 4-month-olds increased their total looking time to the female face in comparison to infants exposed to the control t-shirt [F(1,46) = 5.19; *p*<0.05], but did not change their looking time at the car [F<1]. In sum, while exposed to their mother's body odor, 4-month-olds increased their total looking time to the female face but did not change their looking at the car indicating that the mother's odor enhanced the infants' visual responses to the face.

Finally, our analyses also indicated that the looking behavior of both groups of infants did not differ in terms of the amount of switching between visual targets, latencies to reach any target, or the target that was fixated first (in all cases, *p*>0.05). No other interaction effect with the car picture reached significance (*p*>0.05).

### Looking at the face

**Figure 3:**
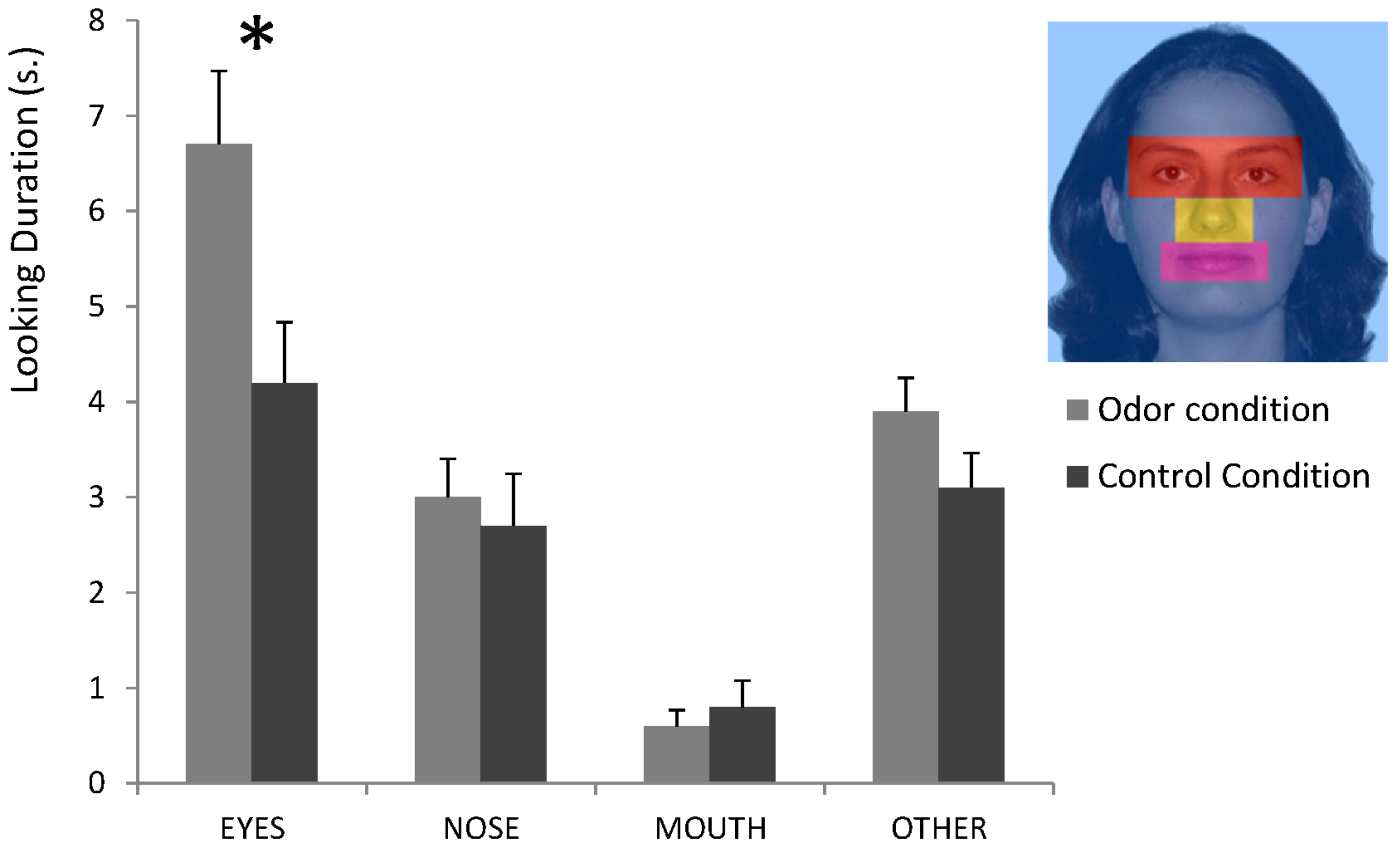
Mean looking duration (in seconds) toward different facial Areas of Interest (AOI; as shown in the insert for the eyes, nose, mouth, and other areas) in a group of infants exposed to a t-shirt carrying their mother's odor (odor group) and in a group of infants exposed to an unworn control stimulus (control group). Error bars represent the standard errors of the mean (SEM). _*_: p<0.05.

A mixed ANOVA with AOI (4), Pairs (2) and Trial (2) as within-subject variables and Odor Group (2) as between-subjects variables was run on the duration of looking directed to the female face only. This analysis yielded a significant main effect of the AOI [F(3,138) = 42.14; *p*<0.0001]. This effect was accounted for by the fact that the infants looked significantly longer at the eye region than at the nose, mouth, or other facial regions (Bonferroni post-hoc tests, *p*<0.0001). In addition, there was a significant AOI×Odor group interaction meaning that looking at the different facial regions was differentially affected by the odor condition [F(3,138) = 3.91; *p*<0.025]. As shown in Figure 3, the looking duration at the AOI covering the eyes in both experimental conditions indicated that the infants in the odor group looked longer at the eyes than did the infants exposed to the control t-shirt (6.7±3.7 *vs.* 4.2±3.1 s, respectively; Bonferroni post-hoc test, *p*<0.01). Thus, it appeared that the increase of overall looking time toward the face in the odor condition reported above was mainly due to an increased duration of looking at the eyes. Analyses were performed to further assess whether this increased looking time to the eyes was due to an increase in the number of looks to the eyes or in the mean duration of each look (*i.e.*, the mean time the infants' gazed within the AOI when they gazed at this region). Despite tendencies for more looks (4.7 for infants in the odor group *vs.* 3.8 for infants exposed to the control t-shirt) and longer means looking times (1651 *vs.* 1145 ms), the difference failed to reach significance [*t*(46) = 1.40, p>0.16, and *t*(45) = 1.63, *p*>0.11, respectively].

## Discussion

The main purpose of the present experiment was to assess whether odors affect 4 month-old infants' visual exploration. Findings indicated that when infants were shown still pictures of a face and a non-social object (a car) they looked longer at the face. In addition, when looking at the face stimulus, infants gazed longer at the eyes than at any other facial region. Both of these expected outcomes differed, however, as a function of presence or absence of a human odor. In the presence of their mother's body odor, infants looked longer at the face and at the eyes. These findings are discussed in more detail below.

### Infants prefer faces and odor strengthens their preference for faces

The development of infant visual responsiveness to human faces has been studied extensively [65]–[66]. Studies have shown that infant interest in faces, as attested by looking direction and amount of looking, is modulated by various perceptual features associated with faces such as their internal configuration, complexity, dynamic nature, familiarity, attractiveness, and/or identity as well as by the cues associated with a person's emotional state, the particular speech s/he produces, and where the person may be looking. Human faces are differentiated from non-face objects from birth onwards [26] and newborns evince a preference for their mother's face over a stranger's face [30], [32]. By three months of age, following extensive face and object experience, infants exhibit a preference for faces over objects ([67]–[68; but see 58]). The present results are in line with the results of previous investigations by showing that 4 month-old infants look longer at a realistic photograph of an unknown human face rather than at a picture of an arbitrary object.

In addition, and most importantly from the current perspective, our prediction that infants' response to faces would be affected by a concurrent olfactory stimulus was confirmed. Specifically, we found that infants exhibited a spontaneous preference for faces and that this preference was significantly enhanced when the olfactory stimulus was a familiar human odor. This result attests to the fact that the maternal body odor carried on the t-shirt was detected by the infants and that it subtly affected their behavior. This is noteworthy because evidence for the sensing of social odors is lacking beyond the neonatal period and before two years of age, even though evidence on responsiveness to arbitrary odors administered on objects or as contextual cues is available for this period in development [19]–[20], [23]–[25], [69], [70]. Thus, in the context of the growing influence of sensory systems mobilized by social interactions (somesthesis, vision, audition) and by own actions (vision, somesthesis, kinesthesis) in the regulation of infant behavior, these data indicate that olfaction is also a salient source of information that directs how other sensory inputs are used in controlling responsiveness in early life.

One interpretation of the effects of a familiar maternal odor on infant looking at faces is that it is mediated by the modulation of attention or interest, linked either to the fact that the odor is in itself arousing (*arousal hypothesis*) or to the fact that concurrent stimulation of multiple sensory modalities (here, vision+odor) produces a redundancy effect [71]. Alternatively, it might be that the odor introduces a familiar cue in an otherwise unfamiliar laboratory setting, making it possible for the infants to more easily recognize their mother or a category of conspecifics (human females), and related familiarity effects may create a context of safety and self-confidence that favor distal exploration through vision (*familiarity hypothesis*). An alternative to the familiarity hypothesis is that infants recognized a familiar odor and detected the incongruence with the unfamiliar face. Although these different hypotheses are reasonable, all of them rely on a general process that should, in principle, increase attention to any visual stimulus, including a face or a car. The fact that the body odor only increased visual attention to the face suggests that none of these hypotheses adequately account for our findings.

The most plausible explanation for the enhancing effects of the odor on looking at the face may be that infants somehow were integrating the olfactory and visual stimulation on the basis of prior experience with similar intersensory links in their daily life. Of course, in the present case, the integration was between a familiar odor and an unfamiliar face, suggesting that by four months of age infants can generalize this form of multisensory responsiveness to novel faces. The fact that they do so in response to human faces but not to non-face stimuli suggests that they associate human odors with human faces and, thus, that odors are interpreted as socially relevant.

### Infants focus on the eyes in a face and odors strengthen that focus

The second important result from this study pertains to the amount of time that infants fixated the eyes. Among all visual features of a face, the eyes appear to be particularly attractive to adults [72], [73]. Importantly, newborn infants also are highly sensitive to the eyes and to their gaze direction [41], [74]. Later, the eyes remain a highly attended internal feature of faces [75], although this general preference is modulated in important ways by whether the face is static or dynamic and whether it is a silent or talking face. When a face is dynamic and talking at the same time, infants begin to shift their attention from the eyes to the mouth but only after six months of life [40]. Thus, regardless of the specific nature of the face, 4-month-old infants focus significantly more of their gaze on the eyes [39], [40], [68], [76], [77]. Our results are consistent with the previous findings regarding young infants' selective attention to different regions of faces and their general preference for the eyes.

Crucially, the current study showed that infants attend even more to the eyes when they are concurrently exposed to the smell of their mother. Thus, a faint odor can make a difference in the way infants look at a face and on which region of this face they focus. In the present case, infants increased their looking at the eyes of a stranger's face. A first possible explanation for this response pattern may be that infants perceived a mismatch between the familiar odor and the unfamiliar face and that they were attempting to extract identity information. If that is the case, the perception of a mismatch between the familiar odor and the unfamiliar face may increase infants' motivation to search for more information about the identity of the pictured conspecific, thus leading to an increase in face scanning (especially of the eyes). This explanation is supported by extensive evidence from adult research showing that facial recognition depends on visual exploration directed at the eye region [87]. A second possible explanation relates to the fact that infants are frequently exposed to the situation where they are held by their mothers and smell her odor while at the same time looking and interacting with other people. So increased infants' interaction with unfamiliar persons in these naturalistic conditions may be related, rather than to a mismatch between the mother's odor and a stranger's face, to the reassuring effect of the familiar odor that prompts infants to allocate more attention and exploration of the physical and social surroundings. The experimental situation here is similar to a naturalistic one (granted the mother is not holding the infants) with the familiar odor floating around them, possibly motivating them to visually explore a stranger's face. Finally, another explanation relates to the infant's expectation of social reward. In everyday circumstances, an infant's perception of maternal odor is generally associated with an interactive face and proximal relations. Further, the familiar odor could support some sort of general arousing effects that promote social attention and expectation even in response to non-familiar people. This may be due to the fact that encounters with strangers usually occur while infants are in close (olfactory) contact with the mother during the first months of life. Accordingly, when exposed to their mother's body odor, it is reasonable to assume that infants increase their attempts to interact with the person in the photograph, especially because by 4 month of age infants have learned that gazing into people's eyes will elicit their responsiveness [78]. Importantly, this behavior is mediated here by specific maternal olfactory cues. The question of whether nonsocial odor cues (e.g., artificial familiar odor cues) might produce similar effects is currently an open one and should be explored in future research.

### Limitations and implications

The present study is limited in that only static and affectively neutral faces were presented. Although infant responsiveness to faces in such conditions may not be generalizable to similar processes in social interactions, their responsiveness was nevertheless clearly different from responsiveness to non-social objects and the looking patterns obtained here were similar to those found in response to dynamic and emotion-laden faces [39], [40], [68]. In addition, even though the faces presented here could be categorized as those of females [62], they were faces of unfamiliar strangers. Finally, even though each infant was exposed to several pictures of faces and cars throughout the experiment, the effect of only one odor was assessed. This might have conveyed several nested perceptual cues. Infants have been found to respond to odor cues of individuality as well as to odor cues of higher-order categorical knowledge [3], [79]. The odor of a person can indeed carry multiple meanings pertaining to individual identity (‘it is my own mother’), or to one or several supra-individual categories related to kinship (‘it is a family member’), gender (‘it is a female’), or gender/age or gender/age/physiology combinations (‘it is an adult female’ or ‘a mother’ or ‘a lactating mother’). Therefore, it would be interesting to investigate in future studies the effects of these various properties conveyed by olfactory stimulation.

The present study may have implications for investigations of perceptual development based on sensory modalities other than olfaction. In many experiments, infants are placed on their mother's, caregiver's, or experimenter's lap during experiments [54], [80]–[83]. In such proximal social conditions of testing, infants may be exposed to multiple stimuli, including olfactory ones, from the mother or other, as well as warmth, touch, movement and noise of respiration, *etc*. Such difficult-to-control social stimuli certainly have the beneficial impact of increasing the social-ecological validity of experiments (see [84]), and hence facilitate obtaining infants' (and parents') compliance with generally unfamiliar laboratory conditions. Although all of these sources of stimulation are typically kept constant across experimental groups, they may nevertheless affect the overall performance of the participants. Here, for example, the mere restitution of the mother's body odor had a measurable, maximizing effect on behavioral variables indexing visual processing.

## Conclusion

In sum, this is a preliminary study examining the impact of olfaction on visual exploration of socially-relevant objects by infants. Prior research has indicated that odors can affect diverse types of emotion-related behaviors in infants (mainly newborns), including arousal regulation, orientation, hedonic appraisal, attenuation of stress- and pain-related responses, and reactivation of memory (references in Introduction). In studying the early behavioral effects of odors in a multisensory context, we found that odors promote increases in visual attention to social objects. Thus, multisensory approaches constitute undoubtedly a promising way to advance our understanding of whether and how olfaction influences human behavior, when in development it plays critical roles in responsiveness, and through which processes it contributes to cognitive processes. In any case, if faces are considered to be “special” because ‘they provide an early channel of communication between infant and caretaker’ [85], at least the same can be acknowledged for conspecific odors. Moreover, the combination of maternal odors and faces provides the strongest “communicative” effect in everyday social exchanges between infants and their mothers.
